# The Use of Immersive Virtual Reality Training for Developing Nontechnical Skills Among Nursing Students: Multimethods Study

**DOI:** 10.2196/58818

**Published:** 2024-07-10

**Authors:** Kitty Chan, Patrick Pui Kin Kor, Justina Yat Wa Liu, Kin Cheung, Timothy Lai, Rick Yiu Cho Kwan

**Affiliations:** 1 School of Nursing The Hong Kong Polytechnic University Kowloon China (Hong Kong); 2 Research Institute for Smart Ageing The Hong Kong Polytechnic University Kowloon China (Hong Kong); 3 School of Nursing Tung Wah College Kowloon China (Hong Kong)

**Keywords:** education, educational, hospital, hospitals, nontechnical skills, nurse, nurses, nursing education, nursing, satisfaction, self-confidence, simulation, simulations, virtual reality, VR, immersive

## Abstract

**Background:**

Immersive virtual reality (IVR) is a niche technology rising in popularity in nursing education. Although there is an abundance of evidence to demonstrate the effect of virtual reality (VR) on desired learning outcomes, this evidence is limited to technical or procedural skills or managing a single patient with clinical problems. Nontechnical skills (NTS), such as communication, decision-making, teamwork, situation awareness, and managerial skills, have not been explored using IVR technology.

**Objective:**

This study aimed to (1) investigate the potential efficacy of the IVR system virtual reality hospital (VR-Hospital, or VR-Hosp), a single-user game we developed, on nursing students’ NTS, sense of presence in the virtual clinical environment, and satisfaction and self-confidence in learning; (2) identify variables that predict NTS; and (3) explore students’ experience in using VR-Hosp.

**Methods:**

A multimethods design with a quantitative and qualitative approach was adopted. Participants were provided with VR-Hosp with 3 scenarios in training. VR-Hosp adopted a multibed, multipatient, multitask approach and was embedded with various clinical situations. Learning outcomes were measured after the training, followed by group interviews.

**Results:**

In total, 202 students joined the study. Results revealed high levels of satisfaction and self-confidence in learning. Significant achievement in NTS was perceived by the students. The levels of satisfaction and self-confidence in learning and the involvement and sensory fidelity domains in the sense of presence were positive predictors of NTS.

**Conclusions:**

The promising results offer a basis for designing IVR activities for nursing education. Further investigations are imperative to determine the impact of IVR technology on learning outcomes in clinical practice.

## Introduction

### Background

Immersive virtual reality (IVR) is a niche technology that has been rising in popularity in nursing education. In the past decade, clinical practice opportunities have declined for nursing students due to personnel shortages and an increasing demand for clinical services [1,2]. Notably, virtual reality (VR) simulations have been recognized for their tremendous potential in nursing education and have shown benefits in performance and knowledge in emergency skills training and single-patient management [3]. In some countries, simulations and other new technology-based training approaches have been accepted as alternatives to replace some of the required clinical hours [4]. Their potential to replace clinical hours became more evident from the closure of clinical venues during the COVID-19 crisis [2,5]. To enable nursing students to develop the competence to solve problems in the clinical context, they not only need to apply the knowledge and skills that they have learned but also need to make decisions when facing situations that they were never taught or had never previously encountered. The question, therefore, is whether VR-based education is a plausible solution to strengthen clinical competence.

### Virtual Reality in Nursing Education

VR is a rapidly expending field, and its definition is complex, ranging from the use of computer-based applications to generate simulated environments depicted on a computer screen to 3D environments with interactive functions and stimuli [6]. VR can be delivered in immersive or nonimmersive modes to establish a varied perception of reality [7]. Examples of nonimmersive modes include online or computer learning and video games. In contrast, IVR education tools or systems are usually delivered using a head-mounted device (HMD) to provide full immersion and interaction in a virtual environment. IVR 3D visualization features make it possible to interact with the virtual environment and offer a deeper sense of presence that distinguishes IVR from web-based or 2D technologies [8]. Bystrom et al [9] and Dang et al [10] have defined a sense of presence as the subjective experience of participants being present within a virtual environment, which is a critical determinant of the level of engagement in immersive learning. Indeed, Dubovi et al [11] found that students’ sense of presence within VR training is positively associated with their conceptual and procedural learning of medication administration. In clinical simulation, a higher sense of presence also allows nursing students to assume a greater degree of responsibility for patient outcomes and reflect on their clinical reasoning and problem-solving skills [12]. We understand the benefits of repeatable training using a simulated environment for building self-confidence and self-efficacy in one’s performance without compromising safety for patients [13]. However, few studies have evaluated the relevance of a sense of presence in the acquisition of nontechnical skills (NTS) in nursing students.

### Nontechnical Skills

NTS are defined as cognitive and interpersonal skills that promote worker safety and complement workers’ technical skills, which include the domains of communication, situation awareness, teamwork, leadership, and decision-making [14]. Traditional health education has primarily focused on the development of clinical knowledge and technical skills, often overlooking NTS [15]. However, increasing evidence links failures in NTS to poor patient outcomes [16]. In multidisciplinary settings, health professionals’ leadership, teamwork, and communication skills are crucial for clinical competence and patient safety [17]. Additionally, in complex and dynamic environments, situation awareness (defined as an individual’s perception, comprehension, and projection of events) and critical thinking (which involves reasoning, deducing, and inducing based on this understanding) are considered essential skills for health care professionals in making effective clinical decisions [18-21]. For instance, an examination of fatal medical accident reports submitted to a third-party safety agency in Japan over a 3-year period found that approximately 50% of these incidents stemmed from failures in NTS, particularly those involving situational awareness, teamwork, and decision-making capabilities [22]. Thus far, most IVR systems have focused on improving knowledge, mastering technical or procedural skills, developing emergency responses, or cultivating soft skills, such as empathy and communication [23-25]. Only a limited number of VR software programs have been designed for learning NTS. Examples of these 2 aspects are task prioritization according to professional guidelines [26] and single-patient management and deterioration detection [27,28]. It is noteworthy that inconsistent results have been found in high-IVR systems for risk perception and safety training in high school students [29]. Systematic reviews have revealed that VR is most effective in improving theoretical knowledge but not affective outcomes and NTS [30-33]. More evidence is needed to substantiate the use of VR technology to prepare nursing students to meet the clinical demands for NTS.

## Methods

### Study Design and Objectives

This study adopted a multimethods design to investigate the efficacy of using IVR via the virtual reality hospital (*VR-Hospital*, or VR-Hosp system (developed by the authors and their team) on developing NTS among undergraduate nursing students. VR-Hosp (short-term patent: HK30083446) is a single-user game that was developed using Unity Pro and HTC Vive Cosmos. Its unique feature of adopting a multibed, multipatient, multitask approach aimed to create a realistic clinical environment with various situations that do not necessarily have a direct relationship to patients’ illnesses. From this, the following research objectives were derived:

- To investigate the efficacy of VR-Hosp on students’ (1) NTS, (2) sense of presence in the virtual clinical environment, and (3) satisfaction and self-confidence in learning
- To identify variables that predict NTS

Qualitative data were collected through focus groups to investigate students’ learning experiences.

### Study Participants and Setting

Participants were undergraduate nursing students in a university in Hong Kong. They were recruited between 2021 and 2022 through convenient sampling from among students taking the “Fundamentals of Nursing” course, a mandatory subject for nursing students.

### Virtual Reality Hospital

#### Conceptual Framework

A simulation model [34] was used to guide the development of VR-Hosp. This model offered a framework to structure the objectives, fidelity, and complexity of the simulation design in relation to (1) teacher and student factors and educational practices, (2) design characteristics and simulation, and (3) outcomes.

#### Teacher Factors, Student Factors, and Educational Practices

The educational practices listed in the simulation model were active learning, feedback, interactions, expectations, diversity in learning, and time spent on tasks. In VR-Hosp, unlike traditional teaching, where learning experiences rely heavily on teachers, VR-Hosp is an immersive VR game with predetermined instructions and game flow. As such, the practice factors are relatively standardized. The activities, time, and criteria for completion were preset based on the learning objectives. Therefore, the expectations were consistent even when the tasks differed. Students were required to make distinctions between and select their actions in response to various tasks to attain the designated goals. Active participation took place since students had to play the game individually and proceed independently. They obtained prompt feedback on whether their actions were correct through answering multiple-choice questions (MCQs).

#### Design Characteristics and Simulation

According to the simulation model, design characteristics relate to objectives, fidelity, complexity, cues, and debriefing. The objective of using IVR in learning is to create a sense of presence that affects learning outcomes. This sense of presence is mapped on to the concept of learning space, as delineated from experiential learning theory [35]. The concept of learning space is that students learn through transactions between the person and the environment. This points to the need for fidelity and complexity in the virtual environment. Learners should be able to subjectively experience their needs, goals, unconscious influences, memories, beliefs, and events, when positioned in the dynamics, interdependence, tension, and forces of the environment.

The fidelity of VR-Hosp was attained through validation of the content and coherence of the stimulus and response elements between the VR and the actual tasks, according to 4 of the 6 principles stated by Harris et al [36]: (1) face validity (whether the VR game looks and feels realistic), (2) physical fidelity (details and realism of the physical elements), (3) psychological fidelity (perceptual and cognitive features of the real task), and (4) affective fidelity (elicits emotional responses, such as stress or fear, in a similar way to the real task) [36]. Construct validity that measures the distinction in performance between novices and experts was not examined at this stage. Ergonomic fidelity was deemed irrelevant since VR-Hosp was not designed to train students in psychomotor skills.

Cues that popped up during the IVR game were essential to motivate and lead the students forward to complete the leaning tasks. In VR-Hosp, such cues were available to guide them. MCQs were also incorporated into the game. If a teacher-guided debriefing session was not available, when students played VR-Hosp individually or with peers, the MCQs would allow them to reflect on their justification for the actions that they performed.

#### Intervention Content

In VR-Hosp, an HMD and a controller held in the right hand were used by players to navigate the 3D virtual ward environment, where there were 3 cubicles, with 6 beds in each cubicle. VR-Hosp was evaluated by a panel of 6 experts, including 3 nursing academics with experience in developing VR games and 3 clinical mentors with rich experience in supervising clinical placements. The game was pretested by another 3 clinical mentors and 2 nursing students for acceptability in terms of content, game instructions, and game flow before it was launched.

VR-Hosp offered 3 scenarios. Each scenario lasted for approximately 10 minutes and comprised 2-3 levels of complexity tailored to students with different levels of clinical experience. The scenarios were named as follows: (1) clinical practicum orientation, (2) managing multiple tasks, and (3) prevention of errors. These 3 scenarios were developed to align with the learning objectives of VR-Hosp: (1) being aware of safety issues, (2) being alerted to contextual incidents/issues in the clinical environment, and (3) prioritizing nursing activities. The 3 scenarios featured the unpredictability of the clinical context with unexpected issues arising randomly. Each scenario within the game provided a context that allowed the students to apply and reinforce the learning objectives. By starting with simpler scenarios and progressively moving to more complex ones, the game aligned with the students’ learning journey, ensuring an appropriate level of challenge and growth.

Each time a player logged in to VR-Hosp, patient deployment, incidents, MCQs, and answer options were generated at random. The VR game used speech recognition and voice recording features (in Cantonese) for students to record dialogues in response to patients’ needs or nurse instructions before implementing care.

The voice-recording feature allowed players to respond to the requests of avatar patients or ward staff. This feature is unidirectional (ie, players must carry out the required actions before progressing to the next step in the game, with the goal of challenging players to critically reflect on the course of actions without any external assistance). Once the players completed the required actions, the MCQs appeared to provide an opportunity for them to reflect on their responses and select appropriate answers. The MCQs were developed based on the principle of reflective learning, facilitating a moment of re-evaluation and critical thinking, reinforcing the learning objectives, and promoting a deeper understanding of the scenarios [37]. In addition, the voice recordings and answers could be reviewed after the VR-Hosp session. By revisiting their interactions, students could, therefore, identify areas for improvement, reinforce their learning, and engage in meaningful discussions during the debriefing.

Based on expert feedback, the response prompts were revised and optimized to provide clearer instructions and ensure a logical progression of clinical scenarios. Revisions to the MCQs were also guided by expert opinions to enhance clarity and alignment with the learning objectives. For instance, to foster critical thinking and promote a comprehensive approach to patient care, the MCQs were refined to simulate realistic dialogues, moving away from standard textbook answers. Moreover, we added answer choices that encouraged players to consider fall risk assessments instead of immediately helping the patient back to bed. The accuracy of the answers was verified, allowing for further refinements. To increase the realism of avatar patients and the clinical environment in VR-Hosp, adjustments were made to the visual and behavioral aspects of the avatars to make them more lifelike and relatable, thereby facilitating realistic patient interactions. For example, in the VR-Hosp simulation, an older male patient exhibiting an unsteady gait was depicted in the ward. His features were adjusted to more accurately reflect the movements typical of an older person. Additionally, to address the issue of VR sickness, we fine-tuned the visual and auditory elements, optimized frame rates, and implemented techniques such as smooth transitions and minimizing sudden camera movements.

**Figure 1:**
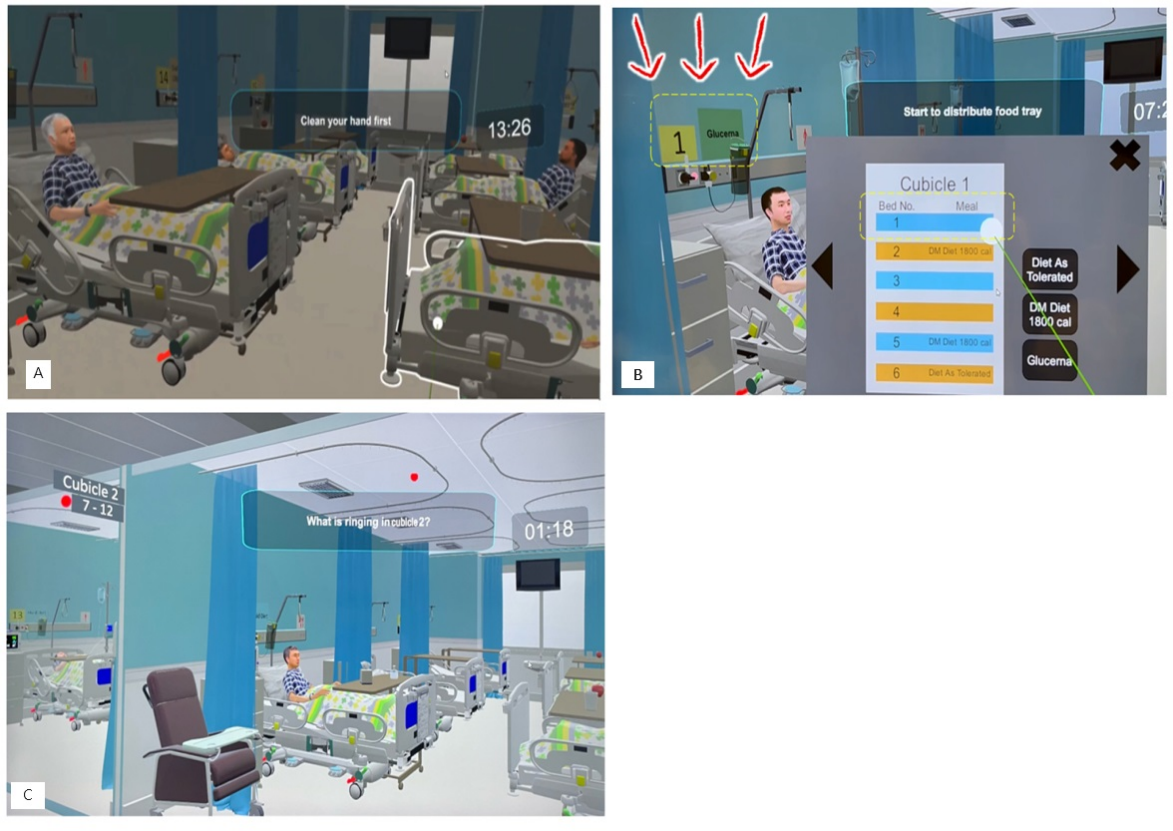
Screen capture of VR-Hosp: (a) cubicle, (b) VR-Hosp environment with a ringing call bell during orientation, and (c) clinical pitfall with inconsistent signage on diet and the meal delivery trolley. VR-Hosp: virtual reality hospital.

Figure 1 displays a cubicle, depicts the VR-Hosp environment with a ringing call bell during orientation, and shows a clinical pitfall with inconsistent signage on diet and the meal delivery trolley. These scenarios require students to be attentive to virtual environments found in clinical settings.

### Procedure

Three sets of VR-Hosp equipment, including HMDs and controllers, and 43-inch televisions mounted on movable stations, were prepared for students to practice on. There were 19 groups of students, with 12-14 students per group. In the first 30 minutes of a 2-hour session, students were given a briefing and practiced operating the controller and the HMD in the VR environment. They were presented with 1 of 3 different clinical scenarios using VR-Hosp to deliver patient care in a hospital ward setting with 3 cubicles. Afterward, they were divided into 3 teams of 4-5 students each and played VR-Hosp on their own. The members of each team took turns at being either a VR player or an observer, while the virtual game was played on the television screen at the same time to enable vicarious learning. The nurse tutor ensured each student in each group had the same amount of exposure to VR-Hosp (ie, 15 minutes), offered technical support on-site, and held a debriefing after each session.

### Measures

The following outcomes were assessed immediately after the VR-Hosp session: virtual nontechnical skills, sense of presence in a virtual clinical environment, and student satisfaction and self-confidence in learning.

#### Virtual Nontechnical Skills

The primary outcomes were 5 personal skills measured using the virtual nontechnical skills (v-NOTECHS) system. Statements in the v-NOTECHS system were modified from the original NOTECHS rating system [14] to facilitate self-reporting on these behavioral parameters during engagement with the VR-Hosp game. The v-NOTECHS system consists of 5 domains: (1) communication and interaction (communication), 3 items; (2) situation awareness and vigilance (situation awareness), 3 items; (3) cooperation and team skills (teamwork), 5 items; (4) leadership and managerial skills (leadership), 5 items; and (5) decision-making, 5 items. One item in the communication domain of the original scale, “waited for acknowledgement from scrub nurse,” was deemed irrelevant in the VR-Hosp learning activities. Players rated the items on a 5-point Likert scale, ranging from 1for strongly disagree to 5 for strongly agree. Cronbach α coefficients in the original scale are between .77 and .87.

#### The Presence Questionnaire Version 3.0

The Presence Questionnaire (PQ) was adopted to measure the players’ sense of presence in this virtual clinical environment [38]. The scale explores how players’ psychological state or attention shifts from the physical to the virtual environment. It consists of 4 domains: (1) involvement (involve—how natural or compelling is it to interact with the environment and control the objects?), 12 items; (2) adaptation/immersion (immersion—how much were you engaged in and focused on the assigned tasks?), 22 items; (3) sensory fidelity (sensory—the degree of coherence for stimulating multiple senses), 17 items; and (4) interface quality (interface—how much did the control or display devices interfere with concentration on the tasks?), 17 items. The highest scores for these 4 domains were 84, 50, 42, and 21, respectively. Note that the items under interface quality were negatively worded. The item scores were reversed so that the higher scores indicated less distraction and delay in the game experience. The respondents gave their ratings using a 7-point Likert scale, ranging from 1 for strongly disagree to 5 for strongly agree.

#### Student Satisfaction and Self-Confidence in Learning

The Student Satisfaction and Self-Confidence in Learning (SSSCL) scale was selected to investigate the design of VR-Hosp [39]. The scale consists of 5 items for the satisfaction subscale (satisfaction), measuring satisfaction with the content and instructions of the game. The second subscale, self-confidence in learning (self-confidence), has 8 items, measured on a 5-point Likert scale. This subscale measures players’ self-confidence in learning associated with the development of NTS. The internal consistency of the SSSCL scale is good. Cronbach α coefficients are .92 and .82 for the satisfaction and self-confidence subscales, respectively.

#### Qualitative Data

An independent senior research assistant who had been trained in conducting semistructured interviews led 4 online focus group sessions. Purposive sampling was used based on the participants’ sociodemographic background (ie, gender, year of study) and whether they had exposure to clinical experience (yes, no) to ensure the representativeness of the focus group sample. Each group consisted of 4-5 participants. Another assistant was present to take notes. The main question posed to the participants was, Can you share your experience with the VR-Hosp game and how it affected your learning? Further inquiries were made about the impact of the experience on their studies; the aspects they liked or disliked; and the skills, knowledge, and other benefits they acquired. Each group’s digital audio recording lasted around 45 minutes and was transcribed word for word for further analysis.

### Data Analysis

Descriptive statistics were computed to show the demographic profile of the students and to capture their self-reported performance in developing their NTS using VR-Hosp. Cronbach α was used to inspect the internal consistency of the questionnaires (α≥.70: good reliability; α=.60: acceptable reliability) [40].

Hierarchical multiple linear regression was used to identify the incremental predictive values of different variables on NTS. In block 1, we aimed to establish baseline relationships by considering the influence of age and gender on NTS, as these sociodemographic characteristics can affect the development of NTS [41,42]. In block 2, we accounted for experience-based factors, specifically prior clinical experience and VR game experience, which not only are associated with the development of NTS but also allowed us to establish their incremental predictive value on NTS beyond the influence of sociodemographic variables [31,43]. Considering a sense of presence has been associated with more positive outcomes in technical skills among nursing students, in block 3, the VR-Hosp game experience measured using the PQ was included to determine its predictive value for NTS, considering the influences from previous blocks [11]. Finally, key predictors in the design of VR-Hosp and confidence in mastering the teaching content using VR, as measured using the SSSCL scale, were entered after considering the contributions in previous blocks. The Technology Acceptance Model (TAM) consists of 3 key components—computer self-efficacy, perceived usefulness, and perceived ease of use—which have been found to positively affect the behavioral intention to learn a health procedure [44]. We used the SSSCL subscale scores because the constructs measured by these subscales closely parallel the components of TAM. For example, the self-confidence subscale of the SSSCL is highly associated with the TAM components computer self-efficacy and perceived ease of use, while the satisfaction subscale is closely linked to the perceived usefulness component [45,46].

NVivo version 11 was used to manage the focus group data. Inductive content analysis was used to examine and analyze the interview data, with the aim of identifying the main categories (themes) in the data and patterns among the subcategories [47]. The unit of analysis was a statement from the transcripts of the focus groups. The exploration and interpretation of the meanings of data led to the emergence of meaningful units of subcategories, and a name was given to each subcategory corresponding to the meanings of its coding. Lastly, the subcategories were condensed to achieve the status of a theme. To ensure trustworthiness, each transcript was analyzed independently by 2 researchers (authors KC and TL), who then met to discuss the data and reach a consensus on the themes [47]. The researchers analyzed the data until they reached the point of data saturation, when no new findings emerged.

### Ethical Considerations

This study was approved by the Human Subject Ethics Subcommittee of the Hong Kong Polytechnic University (approval number: HSEARS20211229002). Informed consent was obtained from all individuals included in this study. Students’ participation was entirely voluntary and would not affect their subject or curriculum in any sense.

## Results

### Participant Characteristics

Of the 237 undergraduate nursing students taking the preclinical VR-Hosp workshop, 202 (85.2%) students consented and participated in the study. Among the participants, who had a mean age of 20.2 (SD 1.45) years (females: n=150, 74.3%), 163 (80.7%) had no clinical experience, while the remaining students had less than 30 days of clinical experience. It is noteworthy that more than 80% (n=167) of the participants had no VR experience prior to VR-Hosp. Sociodemographic characteristics are summarized in Table 1.

**Table 1:** Demographic measures of the participants (N=202) who played the VR-Hosp^a^ game.

Measures		Value
Age (years), mean (SD)		20.2 (1.45)
**Gender, n (%)**		
	Male	52 (25.7)
	Female	150 (74.3)
**Clinical practicum experience, n (%)**		
	No clinical experience yet	163 (80.7)
	Clinical placement for 15 days	27 (13.4)
	Clinical placement for 30 days	12 (5.9)
**Experience in playing IVR^b^ games, n (%)**		
	Never	166 (82.2)
	1-3 years	36 (17.8)

^a^VR-Hosp: virtual reality hospital.

^b^IVR: immersive virtual reality.

### Outcome Assessment

The overall Cronbach α coefficients of the 3 instruments were excellent (Table 2) at .93 (v-NOTECHS system), .95 (SSSCL scale), and .92 (PQ), confirming that the construct was internally consistent (criterion α≥70).The reliability of the v-NOTECHS subscales administered to the target population were satisfactory, with Cronbach α ranging from .70 to .90 (communication=.70, situation awareness=.70, teamwork=.84, leadership=.79, and decision-making=.90). Cronbach α coefficients of the 4 PQ subscales were .90, .85, .80, and .74, respectively. The SSSCL instrument used in the study also achieved a Cronbach α of .93 for the satisfaction subscale and .91 for the self-confidence subscale.

**Table 2:** Outcome measures after participants played the VR-Hosp^a^ game and reliabilities of the scales used.

Outcome assessments		Mean (SD)	Cronbach α	
**v-NOTECHS^b^**				.93
	Communication and interaction^c^	4.3 (0.54)	.70	
	Situation awareness and vigilance^c^	4.1 (0.55)	.70	
	Cooperation and team skills^c^	4.1 (0.55)	.84	
	Leadership and managerial skills^c^	4.1 (0.50)	.79	
	Decision-making^c^	4.1 (0.52)	.90	
**SSSCL^d^ scale**				.95
	Satisfaction with the content and instructions^c^	4.3 (0.56)	.93	
	Self-confidence in learning^c^	4.2 (0.53)	.91	
**PQ^e^**				.92
	Involvement (maximum score=84)	57.6 (8.71)	.90	
	Adaptation and immersion (maximum score=50)	28.3 (4.95)	.85	
	Sensory fidelity (maximum score=42)	39.6 (5.95)	.80	
	Interface quality (maximum score=21)	11.0 (3.39)	.74	

^a^VR-Hosp: virtual reality hospital.

^b^v-NOTECHS: virtual nontechnical skills.

^c^Maximum score=5.

^d^SSSCL: Student Satisfaction and Self-Confidence in Learning.

^e^PQ: Presence Questionnaire.

### Efficacy on Nontechnical Skills

The survey showed that the learning outcomes for NTS were largely satisfactory, with mean scores ranging from 4.1 (SD 0.50) to 4.3 (SD 0.54) out of 5 in the v-NOTECHS scales. In the subscales satisfaction with instructions and self-confidence in learning from the SSSCL scale, mean scores of 4.3 (SD 0.56) and 4.2 (SD 0.53) were also reported, respectively, in the 5-point Likert scale. The sum of the scores for the 4 PQ domains were involvement=57.6, adaptation and immersion=28.3, sensory fidelity=39.6, and interface quality=11.0 (Table 2).

### Predictions of Learning Outcomes

Self-confidence emerged as a significant predictor of 3 v-NOTECHS skills (Table 3): situation awareness (β*=*.21, *P*=.03, adjusted R^2^=0.351, *F*_2,187_=2.084, *P*≤.001), team skills (standardized coefficient β*=*.49, *P*<.001, adjusted R^2^=0.392, *F*_2,187_=39.36, *P*≤.001), and leadership skills (β*=*.31, *P*=.002, adjusted R^2^=0.377, *F*_2,187_=22.32, *P*≤0.001). Satisfaction was documented as a significant predictor of 3 v-NOTECHS skills: communication and interaction (β*=*.34, *P*=.001, adjusted R^2^=0.336, *F*_2,187_=20.01, *P*≤.001) and decision-making (β*=*.39, *P*<.001, adjusted R^2^=0.392, *F*_2,187_=33.44, *P*≤.001).

**Table 3:** Hierarchical regression analysis.

Block of variables			Communication and interaction				Situation awareness and vigilance				Cooperation and team skills				Leadership and managerial skills				Decision-making		
		β		*P* value	△R^2^	β		*P* value	△R^2^	β		*P* value	△R^2^	β		*P* value	△R^2^	β		*P* value	△R^2^
**Block 1**																					
	Gender	.03		.65	0.006	.04		.54	0.004	.09		.20	0.010	.14		.06	0.019	.09		.21	0.008
	Age	.07		.31	—^a^	–.05		.52	—	–.04		.62	—	.01		.93	—	.01		.88	—
**Block 2**																					
	Gender	.04		.61	0.002	.06		.42	0.017	.10		.15	0.016	.14		.06	0.001	.09		.20	0.004
	Age	.07		.39	—	–.03		.76	—	–.04		.16	—	–.01		.88	—	–.01		.93	—
	VR^b^ experience	.05		.52	—	.12		.11	—	.13		.08	—	.01		.87	—	.06		.42	—
	Clinical experience	0		.91	—	–.08		.31	—	–.03		.72	—	.04		.65	—	.02		.81	—
**Block 3**																					
	Gender	–.01		.88	0.186^c^	.01		.89	0.226^c^	.07		.34	0.110^c^	.08		.20	0.208^c^	.04		.51	0.163^c^
	Age	.11		.15	—	.01		.92	—	–.02		.81	—	.02		.77	—	.01		.89	—
	VR experience	.03		.67	—	.10		.13	—	.12		.09	—	–.01		.86	—	.04		.56	—
	Clinical experience	–.02		.78	—	–.08		.29	—	–.03		.66	—	.03		.69	—	.02		.76	—
	Involvement	.30		.02	—	.25		.05	—	.12		.36	—	.18		.15	—	0		.98	—
	Sensory fidelity	.16		.13	—	.27		.01	—	.21		.06	—	.11		.26	—	.22		.04	—
	Immersion	0		.99	—	–.01		.90	—	.03		.81	—	.19		.07	—	.23		.03	—
	Interface quality	–.12		.08	—	–.03		.62	—	–.09		.20	—	–.13		.04	—	–.09		.19	—
**Block 4**																					
	Gender	.03		.59	0.142^c^	.03		.57	0.104^c^	.09		.14	0.256^c^	.11		.07	0.149^c^	.09		.11	0.217^c^
	Age	.09		.19	—	0		.98		–.02		.74		.01		.83		–.01		.87	
	VR experience	.04		.56	—	.10		.08		.13		.03		0		.97		.05		.41	
	Clinical experience	–.04		.54	—	–.09		.16	—	–.06		.35	—	.01		.91	—	0		.96	—
	Involvement	.24		.04	—	.21		.07	—	.07		.54	—	.14		.23	—	–.07		.56	—
	Sensory fidelity	.14		.13	—	.27		.01	—	.21		.02	—	.11		.22	—	.21		.02	—
	Immersion	–.14		.17	—	–.13		.19	—	–.15		.12	—	.06		.56	—	.06		.51	—
	Interface quality	–.05		.44	—	.02		.78	—	–.03		.65	—	–.08		.20	—	0		.94	—
	Self-confidence	.10		.33	—	.21		.03	—	.49		<.001	—	.31		.002	—	.16		.103	—
	Satisfaction	.34		.001	—	.16		.12	—	.08		.41	—	.14		.17	—	.39		<.001	—

^a^Not applicable.

^b^ VR: virtual reality.

^c^*P*<.001.

### Qualitative Data

Basic patterns and coding were clustered and organized into categories (Multimedia Appendix 1). Content analysis yielded 3 categories corresponding to user experience and intended learning outcomes (ILOs). The first category pointed to the fidelity of VR-Hosp. The subcategories were physical fidelity, psychological fidelity, and affective fidelity. The other 2 categories deduced were found to match with the items in the SSSCL subscales and the v-NOTECHS system and, thus, were named satisfaction in learning and development of NTS. The items in the satisfaction subscale indicated the effectiveness of the VR activities in promoting enjoyment and the motivation to learn. Items in the subscale self-confidence in learning pointed to the development of expected knowledge and skills, as specified in the v-NOTECHS system measuring self-reported learning outcomes.

#### Category I: Fidelity

Realism was noted in VR-Hosp.

##### Physical Fidelity

It was observed that not only “the graphics and images were constructed in detail” (student 0207) but also the narrow working space between beds was simulated (student 0304), and “a patient suddenly climbed out of bed and ran really quickly” (student 0310). The chaotic situation in clinical settings was further revealed when discrepancies were noted when the food in the meal cart differed from that indicated in the signage above the patient’s bed (student 1405).

The positive reports from students that VR-Hosp provides a realistic simulation consistent with a hospital environment were corroborated by the quantitative findings of high sensory fidelity scores in the PQ, which measures the degree of coherence in stimulating multiple senses.

##### Psychological Fidelity

Students continually tried to make sense of the chaotic and ad hoc incidents occurring in the virtual environment. Student 0307 mentioned:

I actually experienced the chaos of clinical practice. It feels like what I have learnt was not actually “learnt.”

Another student was stunned by having to create a voice recording in response to the assignment on patients’ needs and nurses’ tasks. The importance of communication became clear, moving the focus solely on psychomotor skills to understand patients’ needs (student 0608).

The unexpectedly immersive learning experience was closely aligned with the level of involvement—specifically, how natural or compelling it is to interact with the environment and control objects. This was particularly evident when students had to create a voice recording in response to an assignment on patients’ needs and nurses’ tasks, prompting them to reflect on the importance of communication in addressing patients’ needs.

##### Affective Fidelity

Tension was reported in the realistic situations embodied in the immersive interactions in VR-Hosp:

In the virtual game, I heard an alarm go off. This would not have occurred in laboratory practice. This made me feel very nervous.

Student 0204

I felt overwhelmed.

Student 0811

These qualitative findings were also corroborated by the adaptation and immersion scores, which assessed how engaged and focused participants were on the assigned tasks. Tensions observed from the realistic and immersive interactions in VR-Hosp offered them a novel learning experience beyond traditional laboratory practice.

#### Category II: Satisfaction in Learning

In this category, students said that this training method was helpful and effective.

Traditional teaching was somehow fragmented, and only focused on a specific area…This VR-Hosp offered us a chance to understand the workflow. In this way, we have learned better.

Student 0706

Not only did they come across various situations that they “did not see in textbooks” (student 1303), but they also had to “analyze information before reporting the patient’s condition” (student 1109). Students enjoyed the learning activities and asserted that the games motivated them to learn. Student 1503 said:

The game that I played was distributing meals to the patients. I did not realize that the meal signage could differ from the actual order.

#### Category III: Self-Confidence in Learning

##### Development of Communication and Interaction

Communication and interaction skills are core components of NTS. Multiple students highlighted how the VR experience helped them realize the importance of these NTS, which they had previously overlooked (students 0608, 0908, and 1306). Another participant also added:

I know communication is important, because I have to respond and find out the priorities of various situation.

Student 0913

##### Development of Situation Awareness

Situation awareness was perceived as a vital skill by many students. They had learned to be observant and alert not only to the environment but also to the patients’ actions to ensure patient safety. As a student mentioned:

Being a nurse, we have to be highly alert since so many different things could happen...What if I did not pay attention and the patient suddenly collapsed?

Student 0410

Another student echoed:

Many a time during laboratory practice, we perform the skills in a step-by-step manner. But in reality, it would not happen as planned. There would be sudden incidents.

Student 1312

##### Development of Decision-Making

Decision-making was 1 of the central learning outcomes. Knowledge and clinical reasoning all came into play. Moreover, prioritization was deemed “essential since tasks came up one after another” (student 0710). “[We] have to judge by ourselves” (students 0105, 0211, 0510, and 0907) was a comment that was made many times. In addition, students said, “We needed to determine the priority” (student 1301), learned to “analyze the information” (students 1008 and 1506), “think critically” (student 1401), and “know the rationale for our actions” (student 0311).

Student 1004 pointed out that “it felt so real that you would be asked to do another thing while you are busy.” Students also found that they needed to “multitask” (student 1403) and that “there is an internal timer” (student 0713). One student best summarized the experience of playing VR-Hosp:

We had to be efficient, accurate, and careful.

Student 0406

## Discussion

### Principal Findings

This study was the first of its kind to explore nursing students’ experience in using the immersive game VR-Hosp to learn NTS. Overall, findings suggested that VR-Hosp has the potential to facilitate NTS learning in order to complement current educational strategies.

Both quantitative and qualitative findings indicated the positive effects of VR-Hosp training in enhancing nursing students’ NTS through a high-stress, time-critical IVR environment that customized real-life clinical situations with multitasking and episodes of interruption, demanding heightened awareness and prompt decision-making. Sensory fidelity signified the realism and coherence of the senses, for example, sound and movement, and the examination of virtual objects from multiple viewpoints. Huang et al [48] found that a high sense of presence might generate a cognitive overload when individuals are trying to complete virtual tasks, and thereby negatively affect satisfaction. In contrast, visual, auditory, and tactile stimulations were found to be vital for novice nurses to detect cues related to patients’ conditions [49,50]. In a similar vein, sensory fidelity in VR-Hosp allowed the students to play individually with high concentration and meticulous cognitive and perceptual responses, to critically think, and make appropriate decisions. Consistent with another study, sensory modalities to imitate real-world movements were crucial in learning [51].

In our quantitative findings, critical sensory input was found to be associated with perception and comprehension of the situation [52]. More importantly, the meaningfulness and coherence of the content and activities were established as factors that promote learning and the goals of higher education [53]. The high satisfaction and self-confidence scores, coupled with their predictions of these 2 NTS, unpacked the meaningfulness of using VR-Hosp. These findings were further complimented by students’ feedback, which indicated that the virtual environment realistically offered space for them to make decisions and react to the visual or auditory alarms, instead of providing a step-by-step guide. Students were required to multitask within a set period and to tend to episodes of sudden demands. Prioritization and communication with the patient or other members of the health care team were significant. Students had to contemplate the rationale for their actions and reconsider their justifications when answering the MCQs in response to the virtual situations. Hence, the IVR activities facilitated the building of situation awareness and vigilance, as well as decision-making.

The qualitative findings provided further insights into the distinct contributions of the game’s design to the development of NTS among nursing students. Detecting and handling alarms, hazards, and clinical pitfalls were the learning activities in VR-Hosp. Students commented how VR-Hosp heightened their excitement, eliciting stress and nervousness when encountering unexpected incidents and equipment alarms during the game play. It was uncovered that the voice-recording feature in VR-Hosp made the students feel compelled to communicate with the nurse and patient during the game. They could not proceed to the next action if they did not talk to the patients and other health care workers. Such forced interactions urged them on to communicate with the avatar and engaged them in performing the designated activities. Altogether, these qualitative findings echoed the learning space concept in the experiential learning framework [35], contributing evidence of VR-Hosp’s sensory fidelity value to physical, psychological, and affective fidelity.

Previous studies have reported that gender and experience in playing VR games are factors that affect satisfaction and usability scores [23]. In contrast, our quantitative findings revealed that gender and literacy in VR technology do not have an impact on situation awareness and decision-making. It was interesting to discover that the other 3 domains in the sense of presence did not predict either situation awareness or decision-making. These domains were involvement (controlling and moving in the virtual environment), immersion (proficiency and consistency with the real world), and interface quality. This means that interference in using control devices, delay in experiencing actions, and distraction in visual display did not hinder students’ confidence in their ability to develop the desired skills. It was inconclusive whether naturalistic interactions are the element that influence how the form and content of the learning modalities operate in virtual learning environments [54]. That said, it was likely that exposure to IVR clinical practice was significant in helping novice nurses develop and master the skills of situation awareness and decision-making regardless of the control of VR devices.

Overall, this study offers evidence of the sensation fidelity of the VR environment as an essential feature to achieve learning outcomes. Our findings suggest that VR-Hosp has the potential to facilitate the development of situation awareness and decision-making, complementing current educational strategies. For instance, IVR provides a cost-effective and accessible alternative to traditional pedagogy, such as high-fidelity simulation. Although high-fidelity simulation is an effective educational strategy, certain limitations, such as shortages of personnel, resources, and space and the lack of available qualified facilitators, can impact its implementation and effectiveness. However, IVR eliminates the need for physical resources and a dedicated space, allowing students to engage in realistic scenarios using VR headsets or other devices. This scalability enables a larger number of students to participate simultaneously, enhancing accessibility and reducing logistical constraints. With the use of IVR, facilitators can guide and debrief students, leveraging the recorded interactions and performance data to provide targeted feedback and facilitate reflective learning.

### Limitations

This study adopted a convenience sampling method; thus, it is difficult to generalize the results. The study involved a cross-sectional survey collected after VR-Hosp practice; thus, the cause and effect of this VR teaching strategy could not be determined. NTS in the real world are often influenced by external factors, such as high uncertainty and time pressure [55]. Considering that v-NOTECHS scores were self-reported, further work is needed to objectively evaluate the learning outcomes and assess whether these skills can be sustainably translated to realistic settings. When implementing VR-Hosp, students took turns in being players and observers. This might have interfered with the immersive experience since the observers communicated with the players during the activity, for example, in locating alarms and when answering MCQs. There might also have been bias when obtaining qualitative feedback, since the process was conducted during the debriefing session moderated by the teachers. However, the information that was obtained forms a basis for future studies to compare its impact with that of other educational pedagogies. In addition, the use of HMDs in IVR can lead to VR sickness, such as nausea, dizziness, and blurred vision [56]. Although our study did not report cases of VR sickness, its presence could negatively impact the immersive learning experience. Future research should include measures such as the Virtual Reality Sickness Questionnaire to evaluate its effect on the desired learning outcomes [57]. Additionally, future studies using IVR may consider striking a balance between realism and incorporating elements shown to reduce VR sickness, such as narrowing the horizontal field of view, partially limiting the degrees of freedom in navigation control, and increasing tactile feedback [58].

### Conclusion

VR-Hosp appears to be a promising educational pedagogy for enhancing NTS, including situation awareness and decision-making ability, in nursing students. VR-Hosp portrays a nonlinear world that challenges students to operationalize what they have learned in traditional classroom teaching and simulation practices. The findings add evidence to the determinants of learning outcomes from the aspects of a sense of presence, satisfaction, and self-confidence in learning. This should motivate the undertaking of future work on VR-based teaching and learning activities in higher education.

## Appendix

Multimedia Appendix 1 Content analysis: overview of categories and subcategories.

## Abbreviations

HMD: head-mounted device
IVR: immersive virtual reality
MCQ: multiple-choice question
NTS: nontechnical skills
PQ: Presence Questionnaire
SSSCL: Student Satisfaction and Self-Confidence in Learning
TAM: Technology Acceptance Model
v-NOTECHS: virtual nontechnical skills
VR: virtual reality
VR-Hospital/VR-Hosp: virtual reality hospital
