# Supplementary material for: The Use of Immersive Virtual Reality Training for Developing Nontechnical Skills Among Nursing Students: Multimethods Study
Source: Asian Pac Isl Nurs J. 2024 Jul 10;8:e58818. doi: 10.2196/58818 (PMC11269964; doi:10.2196/58818)
Supplement: Multimedia Appendix 1 [file apinj_v8i1e58818_app1.docx]

**Table S1.** Content analysis: overview of categories and subcategories.

| Categories and subcategories | | | Selected sample quotes |
| --- | --- | --- | --- |
| Fidelity | | | |
|  | | Physical fidelity (detail and realism of the physical elements) | - “The experience is fun. The graphics and images were constructed in details” (student 0207). - “Learn in a realistic situation. Before playing the VR^a^ game today, I did not realize that one nurse has to take care of so many patients. I cannot believe there are so many beds, and the space between beds are so narrow, which barely allow one medicine cart to get through” (student 0304). - “There are a lot of situations. When we play the game, each one of us will face a different situation. In my case, a patient suddenly climbed out of bed and ran really quick, and said he wanted to make a phone call. I chase after him. I felt such things could have happened in real settings. So it is a very special experience” (student 0310). - “It is easier to control the VR device than what I thought. For example, it is easy to control the meal cart and the dressing trolley. Other students measure blood pressure when they played, but I distributed meals in my turn. I have never thought of what I would have experienced! I should have checked the overhead signage and double confirm the meal before I offer it to the patient” (student 1405). |
|  | | Psychological fidelity (replication of the perceptual-cognitive demands of the real task) | - “I actually experienced clinical practice and was in chaos. It feels like what I have learnt was not actually ‘learnt’, and I seemed to know nothing and do not know what to do. I needed to handle so many things, and I felt I am now psychologically prepared for later clinical practicum. It is so important that I have to revise my books” (student 0307). |
|  | | Affective fidelity (elicit emotional responses, such as stress or fear, in a similar way to the real task) | - “In the virtual game, I heard alarm went off. This would not have occurred in laboratory practice. This made me feel very nervous” (student 0204). - “I had to work independently in the entire ward, and I felt overwhelmed” (student 0811). |
| Development of NTS^b^ | | | |
|  | Communication and interaction | | - “It was really fun to play the VR game. Perhaps I only focused on the skills in my usual practice. I now realized that I have to communicate to the patients to let them understand what is going on” (student 0608). - “I think it is really good that we have to voice record, and communicate with the nurse or the patient. We seldom have anyone talking to you when you practice” (student 0908). - “I know communication is important, because I have to respond and find out the priorities of various situation” (student 0913). - “In the past, we only learn about the skills. In this VR game, we practice in realistic scenarios. We have to talk to the nurse in charge. In general, that is something you were not taught in the class” (student 1306). |
|  | Situation awareness | | - “Being a nurse, we have to be highly alert since so many different things would happen…What if I did not pay attention and the patient suddenly collapsed?” (student 0410) - “We need to be careful. I need to respond immediately when a patient choked. Many a time during laboratory practice, we perform the skills in a step-by-step manner. But in reality, it would not happen as planned. There would be sudden incidents” (student 1312). |
|  | Decision-making | | - “It is fun. I just measured the blood glucose. I have to analyze the information. After I had made the wrong conclusion twice, I picked up how I could act appropriately according to the different blood glucose values” (student 1506). - “When I went to the nursing station and talked to the nurse, she asked me to perform a task. However, we do not just follow what was told, but we have to judge by ourselves. It is close to the realistic situation. It is fun” (student 0211). - “After playing the VR, I realized that a nurse have to handle numerous tasks. While you were taking blood pressure, suddenly you were asked to perform a wound dressing. We have to justify and prioritize instead of following the routine” (student 0510). - “I played scenario 3, and administered the wrong medication. Often times, I realized that there would not be someone guiding you all the time. If your knowledge is deficient, you cannot help others. As such we made mistakes. Now I realized, to be a nurse, we have to do better. Therefore, I have to be knowledgeable” (student 0407). - “I learnt how to communicate with other colleagues. Apart from finishing my job at hand, I have to take up newly assigned tasks. A nurse at the nurse station would suddenly asked you to perform wound dressing when you are taking blood pressure. Multitasking was needed, and you have to prioritize the tasks” (student 1403). - “It felt so real. You have to finish some task with a set time. It reflected that we have to be practice more so as to be more proficient. Prioritization were deemed essential since work comes one after another” (student 0710). - “I just finished scenario one. I could not say the tasks were very difficult. However, we had to be efficient, accurate and careful. Hence, when an alarm went off, you have to walk around and observe for what happened. You have to be aware even a bed was not locked, otherwise that patient would be endangered” (student 0406). - “I felt that the VR game is so different from traditional classes. There would not be a load of task to perform. However, today, I went to the VR ward, there are heaps of tasks. You will have to memorize what to prepare and do aftercare. Moreover, although no one really time it for you during your work, but there is always an internal timer” (student 0713). - “It felt realistic. A senior nurse would ask to do another thing while you are busy. You have to explain to them the situation” (student 1004). - “We had to familiarize with the situation. In general, there were numerous tasks for you to perform. We have to judge and prioritize according to our rationale. The workflow should be more smooth” (student 0907). - “Like realistic situation, it is not a must to perform a specific task in a set sequence. We can have our own judgement” (student 0105). - “When sudden issues arose. For instance, while you were performing a wound dressing, sudden a call bell rang. We needed to determine the priority how you proceed to manage these situations” (student 1301). - “I felt very good. I did not have any clinical practice experience. This is a good chance to learn. We need to complete a lot of tasks in 10 minutes. I have to plan and priorities the tasks. I trained my critical thinking” (student 1401). - “I have to check the record prior to blood sugar measurement, and analyze the information. This helped decision making in the next step” (student 1008). - “The activity is interesting to me. I have to change bed sheets and perform wound dressing. This is when I need to understand the priorities between these two tasks. At first, I thought it doesn’t matter. However, there is a reason why you made a certain choice. Next time when I practice in the ward and were asked to perform another task in the middle of my work, I knew the rationale of our actions. How to respond was also another skills to learn” (student 0311). |
| Satisfaction in learning | | | |
|  | Training method helpful and effective | | - “Traditional teaching was somehow fragmented, and only focused on a specific area…This VR-Hosp^c^ offered us a chance to understand the workflow. In this way, we have learned better” (student 0706). - “I encountered various situations that was not seen in textbooks. For example, I was asked to take care of a patient who was vomiting” (1303). - “I could try many tasks, wound dressing, etc. After analyzing the information, then I have to report the patient’s condition” (1109). |
|  | Enjoyed the activities | | - “It was fun! I realized that we have to do the right assessment and care to the right patient” (1503). |

^a^VR: virtual reality.

^b^NTS: nontechnical skills.

^c^VR-Hosp: virtual reality hospital.
